# Healthcare professionals’ experiences of video consultations in palliative care in rural areas: an intervention study in community care

**DOI:** 10.1186/s12913-024-11196-5

**Published:** 2024-06-17

**Authors:** Margareta Brännström, Johan Philipsson, Sofia Andersson

**Affiliations:** 1 Department of Nursing, Umeå University, Skellefteå, Sweden; 2 Cancer Centrum, Region Västerbotten, Sweden

**Keywords:** E-health, End-of-life care, Palliative care, Qualitative research, Rural care

## Abstract

**Background:**

The population is aging, leading to an increased need for palliative care and end-of-life care. There is a lack of research on the use of video consultations for knowledge transfer between specialist and general palliative care. The aim of this study was to describe healthcare professionals’ experiences of video consultations in palliative care in community homecare and nursing homes in rural areas.

**Methods:**

Individual interviews (*n* = 11) were conducted with five community nurses, one occupational therapist, two specialist palliative nurses, and three specialist palliative care physicians. The data were analysed using reflexive thematic analysis.

**Results:**

The analysis identified three themes: *feeling comfortable with increased availability of specialist expertise*; *seeing each other facilitates communication*; and *being supported by physically present care professionals is essential*.

**Conclusion:**

HCPs suggest that video consultations are an effective way to increase access to specialist palliative care and provide more equal care to patients with palliative care needs in rural community care.

**Supplementary Information:**

The online version contains supplementary material available at 10.1186/s12913-024-11196-5.

## Background

The population is ageing, leading to an increased need for palliative care and end-of-life care. Palliative care is based on a holistic approach and is intended to improve the quality of life of patients with serious illness and their family members [1, 2]. Patients with complex symptoms should be offered specialist palliative care according to the national care program in palliative care in Sweden [3]. According to Sweden’s National Board of Health and Welfare [4], the challenge is that many persons require specialist palliative care at end of life, but access is limited and unequal across the country. A study using data from Sweden and other western European countries demonstrated that access to palliative care is far from equal even in countries in western and northern Europe [5]. A review showed that most patients needing palliative care who live in rural areas wish to be cared for at home for as long as possible [6]. According to the Swedish Register of Palliative Care [7], access to specialist palliative care is determined by population density, distance, availability of trained healthcare professionals (HCPs), and medical conditions.

The Ministry of Health and Social Affairs [8] issued ‘Vision for eHealth 2025’, calling for Sweden to be a pioneer in healthcare digitalization to enable good and equitable healthcare and well-being. In the associated project, eHealth in palliative care is limited to real-time video consultations. A review has shown that virtual care, particularly when used to complement rather than replace in-person meetings, yields similar or even better results in terms of quality of life. The review results also showed that virtual care modalities are both safe and effective in end-of-life and palliative care, without any observed negative consequences [9]. A mixed-method study from Ireland showed that patients were overall satisfied (93%) with video consultations used in discussing physical needs (90%), medications (90%), and psychological symptoms (83%). A minority (16%) of these patients expressed dissatisfaction that they could not be physically examined. Additionally, only a minority (37%) were satisfied with discussing spirituality and faith in the consultation [10].

In an intervention study from Australia, specialist palliative care physicians were integrated with community nurse visits to patients’ homes. The results indicated the effective expansion of integrated specialist palliative care support to rural communities and the acceptance of this care among patients, caregivers, and HCPs. The results also showed that the telehealth model optimized health resource utilization during the final 30 days of life, offering promise in reducing distress in this critical period [11]. A brief report showed that palliative care video visits in nursing homes produced perceived benefits by enhancing communication among the participants. These visits facilitated remote family engagement, provided visual insight into resident status, enabled discussions of goals and preferences, and offered flexibility in adapting to the nursing home environment [12].

Qualitative studies from Denmark indicate that video consultations with specialist palliative care can improve the quality of palliative homecare. It was also found that collaboration between the palliative care team and the community nurses was improved [13, 14] and that patients and their families were more engaged [14] through video consultations. A pilot study from Canada involving patients, loved ones, and HCPs suggested that video consultations could be an effective and feasible method to bridge geographical distances [15]. A brief mixed-method report showed that clinicians (both physicians and nurses) rated video visits as primarily useful for follow-up visits, that physicians felt competent providing some services through video, and that they had generally positive attitudes toward video visits and telehealth. The interview findings further highlighted that clinician enthusiasm may be tempered by technological issues [16].

A review revealed a lack of research on video consultations between specialist and general palliative care, and for conditions other than cancer; the review also showed that there were no relevant studies from Sweden [17]. Another review [6] included only one study from Sweden, indicating a scarcity of research in the Swedish context, Palliative care is primarily provided by general health care professionals with palliative care experts in consulting roles, and sometimes palliative care experts take over care for the patients. The latter literature review demonstrated a lack of research on video consultations involving specialist palliative consulting teams in community homecare and nursing homes, encompassing perspectives of patients, families, and HCPs.

### Aim

The aim of this study was to describe HCPs’ experiences of video consultations in palliative care in homecare and nursing homes in rural areas.

## Method

### Design

This study was a descriptive qualitative study. Semi-structured interviews were conducted with healthcare professionals. The consolidated criteria for reporting qualitative research (COREQ) were used to report the study [18].

### Study context

This study was part of an intervention project addressing the provision of specialist palliative care to patients, family members, and HCPs via video consultation in homecare and nursing homes. The research context was a small municipality in northern Sweden with approximately 7000 inhabitants.

### The intervention

The palliative consulting team (PCT) at the cancer centre of the hospital is a supportive resource that provides specialist palliative care through counselling, supervision, and education to healthcare professionals, patients, and family members. The intervention was done through video consultations and video-based communication support (using tablets and laptops), connecting patients with the PCT for conversation, assessment, and guidance. The PCT consisted of a nurse and a doctor who coordinated with the primary care doctor on duty, the community home healthcare team, staff within the community’s home healthcare and nursing homes, as well as patients and family members in the patient’s homes. The palliative care registered nurses (PCRN) have experiences of using video-based communication support. Planned video consultation (weekly) were conducted with the PCT and community care staff. In some video consultations, the physician participated through video; in other situations, the physician specialised in palliative care was physically in the room with the patient while the family members participated through video. Some video consultations occurred between the community registered nurses (CRNs) and the PCT.

### Participants

All participants in the intervention were asked to participate (*n* = 12). A total of 11 HCPs with experience with video consultations in specialist palliative care participated in the study, comprising CRNs (*n* = 5), an occupational therapist (*n* = 1), PCRN (*n* = 3), and physicians specialised in palliative care (*n* = 2). One invited general physician declined to participate in the study. Most of the participants were female (*n* = 10) and the mean age was 45 years.

### Data collection

Qualitative data were collected in autumn 2022 and spring 2023, interviews were conducted individually both face to face at workplace, and through Zoom due to the COVID-19 pandemic (median 20 min, range 12–30 min). The interviews were digitally recorded and transcribed verbatim. All interviews were conducted by two of the authors (JP, SA). JP is a PCRN who is well experienced in palliative care, SA is a scientist, who has extensive experience in qualitative research and interviewing. An interview guide was used and included questions about the participants’ backgrounds and experiences of video consultations. The opening question was: ‘Can you tell me about your experiences of video consultation with patients?’ Clarifying questions were asked, such as: ‘How did you feel about that?’ and ‘Can you tell me more about that?’ The interview guide used in our study was developed for this study (supplementary file).

### Data analysis

The interviews were analysed by reflexive thematic analysis approach inspired by Braun and Clarke [19, 20]. Initially, two authors (MB, SA) thoroughly read the entire dataset. During this process, initial codes were generated through an inductive approach by the first and last authors individually. These codes were then examined and refined, after which we organized these codes into coherent themes. Once the themes were well-defined, we revisited them to ensure that they were supported by the data and that there was no overlap between them. After this review, the themes were finalized.

## Results

The analysis is presented according to three themes: *feeling comfortable with increased availability of specialist expertise*; *seeing each other facilitates communication*; and *being supported by physically present care professionals is essential*. The results are illustrated with relevant quotations.

### Feeling comfortable with increased availability of specialist expertise

Video consultations were described as time saving for both patients and HCPs because they required less travel and were not weather dependent. They were also described as enabling more equal care and saving patient energy. It was important to be prepared and have technical skills for the video consultations, otherwise they could be stressful for the CRNs. Hindrances to video consultations were described, such as poor access to Internet connections in sparsely populated areas.

CRNs described a need for better knowledge of palliative care. Video consultations increased the availability of specialist palliative care and were described as supportive. They were also described as allowing fast access to meetings with specialist physicians and Registered Nurses in palliative care or other specialities, such as pulmonology and oncology. The CRNs emphasised that video consultations access to specialist palliative care was positive and has facilitated improved homecare quality. The importance of offering support was highlighted by PCRNs.

So it’s not always that we [i.e., PCRNs] have concrete advice to offer, but at the very least, being able to confirm that what they are doing is good provides, like, collegial support. I believe that is valuable for those you are talking with, and it becomes valuable for oneself as well to receive … support in thoughts and such. (participant 8)

The CRNs described being able to learn from the PCT, which was useful for providing collegial and professional support. The participants discussed and helped one another clarify patient needs and find options for actions and solutions. The CRNs could show, for example, wounds and technical equipment using the laptop, in turn receiving good advice about treatment. They felt supported when they could discuss difficult situations, such as giving or not giving treatment, drug doses, and use of support stockings:

Yes, and then there could be someone who had worries and anxiety—well, how much, yes, then the frequency of Midazolam (sedative), how often should we administer it? (participant 7)

### Seeing each other facilitates communication

It was considered an advantage to meet via video consultation because it let the participants see one another, in comparison to phone calls. When the technology worked well, the participants felt as if they were in the same room as the patient. It was described as nice to see the person one was talking with, as it made the exchange more personal. The participants said that it was easier to build relationships with patients through video consultation because they could see one another and interact on the same ‘wavelength’, in comparison to phone calls. Video consultation enabled more people to participate in the conversations. The HCPs also organized team meetings through video consultation. The CRNs´ said that the PCT was responsive and would listen, and that it was easier to explain things when they could see one another:

Yes, everyone is here, the PCRN, plus the doctor and the nurse behind the screen – it really creates this sense of teamwork, you know, especially in this interprofessional setting. (participant 9)

The CRN emphasized the significance of involving patients and their family members in video consultations, whether they were located locally or in different regions, including other countries. Establishing a relationship and fostering a shared understanding of the patient’s care requirements and everyday situation were essential. This was particularly significant, for instance, when patients wanted to discuss end-of-life matters:

We had a grandchild running in and out of the screen, and she even participated in the conversation, adding joy to the whole situation. So [the doctor] even said, ‘It’s great to have a grandchild to remind us that life goes on’. (participant 7)

The CRNs´ highlighted that broaching the topic of imminent death during a video consultation frequently brought about discomfort. However, there were situations in which postponing the conversation was not a viable alternative. It is important to inquire about the patients’ priorities and facilitate their ability to express their own feelings and thoughts:

But to deliver the very first news [of imminent death], I don’t find it appropriate, but to reinforce and clarify [the situation], I see no obstacle to doing so. (participant 6)

### Being supported by physically present care professionals is essential

During video consultation with specialist palliative care, it was described as essential for a community HCP to be physically present for the patient and the family members. It was important to provide physical touch to provide affirmation – ‘I felt like an extended arm’. The CRNs who were physically present described having the opportunity to support the patient, for example, touching the patient and offering the patient a napkin. They could also examine wounds and symptoms. The PCRNs valued this as an opportunity for complementary action because they were not physically in the same room:

I’m thinking that we were there and could give comfort, well, maybe touch him or, well, a bit like she might have done if she had been present. (participant 4)

The CRNs who were physically present described having the opportunity to read the patients’ non-verbal communication, such as body language and facial expressions, to a greater extent. They emphasized that they acted as the patients’ ‘mouthpiece’. It was important not to leave patients and family members alone to receive difficult news and have difficult conversations via a video link: there should be someone present during challenging discussions, for example, about ending cytostatic treatment. Nothing was described as inappropriate to address in video consultations, but the importance of knowing what information patients had previously received was emphasized. It was also important to prioritize in-person visits if patients had not received certain sensitive information before:

To be informed about imminent death, such as “You are seriously ill” or “This won’t end well,” is hopefully something that the patient is informed about before leaving the hospital. Many receive that information at least before going home, although it may not apply to everyone (participant 1).

The PCRNs said that it was difficult to sense the atmosphere in the room and read the patient’s body language and facial expressions. The PCRNs also mentioned that they used other communication tools to more clearly detect, for example, the patients’ feelings:

I think in that case you need to be a bit sensitive to these subtle cues, perhaps body language, and clarify like, ‘It seems like you’re …?’ or ‘Was this a surprise for you?’ In other words, you may need to verbalize and help interpret body language if it’s not picked up by the person on the other side of the screen. (participant 6)

The CRNs´ thought that there were some situations in which it was important for the patients and family members to talk with the physician alone, and therefore would offer to leave the room. Decreasing the number of people in the room could be a way to help the patients to open up.

The nurses said that if the patient had a hearing, visual, or cognitive impairment, it could be challenging to communicate via a video link. On the other hand, a CRN and a PCRN described a video consultation with a patient with visual impairment who could sense that the nurse (PCRN) was in the room:

Then I was physically present, and both the patient and the patient’s husband were present. However, the rest of the family, many children, were connected online. So, there was the patient on site, along with family members through a link, and it was actually quite good, even though it was a bit stressful at first. (participant 9)

## Discussion

This study found that video consultations in palliative care in homecare and nursing homes was experienced as positive by healthcare professionals. Our findings suggest that video consulting in palliative care can contribute to more equal access to care in rural community care. It gives patients the opportunity to have closer and more frequent contact with specialists in palliative care, not just in conversation but also via showing them their physical symptoms, such as wounds. Lundereng et al. [21] described telehealth as enabling healthcare professionals to remotely note patients’ visual status, surroundings, and even emotional status. In our study, video consulting was also found to permit the participation of other specialists in the care of the patient. One core finding was that the CRNs felt that they received collegial and professional support from the PCT, increasing their level of knowledge. That support included having the opportunity to discuss both symptom management and more ethical considerations, such as difficult situations near end of life. By involving staff from other units, Perrin and Kazanowski [22] argued that palliative consulting improved the quality of life of patients as well as making staff more comfortable with caring for these patients.

In our study, healthcare professionals considered video consultations a better option than phone calls. Enabling all participants in a consultation to see each other, albeit through a screen, enhances the conversation and strengthens the relationships between the patient and/or family members and the healthcare professionals. Lundereng et al. [21] described healthcare professionals feeling more connected with patients and their families when using video than the telephone.

Using videoconferencing in home-based palliative care offers knowledge of the patient’s surroundings, making the consultation more personal [21]. In our study, the video consultation facilitated the involvement of family members, helping clarify the patient’s care requirements. Vestergaard et al. [23] found that including family caregivers in video consultations in the hospital adds a family perspective and helps the close family members contribute important information about the patient. They also saw it as a flexible way for family caregivers to participate in patient care during the patient’s hospital stay. Our study indicates that some topics are less suitable to discuss in video consultations, for example, prognosis, and new information about serious illness or imminent death. However, communication about serious illness should be done in person, which was deemed more appropriate. Nevertheless, there may be no other opportunities to discuss such matters. Lundereng et al. [21] found that the usefulness of telehealth depends on all participants already having trust in each other, suggesting that video consultations are more appropriate for follow-up, and initial contact is preferred to be face-to-face.

An essential finding of this study is the value of having a community HCP physically present with the patient and family members during the video consulting. The CRN acted like an extended arm of the PCRN in multiple ways, partly by being present, helping with the patient (e.g., examining symptoms), but also by ‘reading the room’, picking up details of the interaction among those involved in the video consultation, and helping mediate this to the PCRN. A systematic review by Engel et al. [24] showed that healthcare professionals can make patients and family members feel welcome and secure in expressing their experiences, concerns, and needs and in sharing their goals and preferences for treatment and care. Patients and family members want healthcare professionals to align themselves with their process of assimilating and coping with information because honest information could induce fear, stress, and existential disruption. In communication with healthcare professionals, they have their own dynamics; healthcare professionals must align themselves with them, crucially guiding patients and family members through their information assimilation process over time. Communication itself is complicated and requires experience and training. Video consulting has often been used as a replacement for physical present care professionals. We found that being supported by physically present care professionals is essential, and initial contact is preferred to be face-to-face. Video consulting increases difficulties in the information assimilation process. To compensate for the potential shortcomings of video consulting among patients, their families, and PCRNs, in the case of reading nuances in communication and being able to conduct physical examinations, we see great benefits in having a healthcare professional physically present with the patient during the video consulting.

There was a small number of participants in this study. However, the interviews were rich in content, and the participants were eager to narrate their experiences. Trustworthiness in a qualitative study is gained more by the richness of each interview than by the sample size [25]. We tried to describe the analysis thoroughly and verified the themes with original quotations from several participants. To ensure credibility, the research team described the intervention’s context and the participants’ characteristics. It is important to highlight general practitioners’ experiences in further studies.

## Conclusion

HCPs suggest that video consultations is an effective way to increase access to specialist palliative care and provide more equal care to patients with palliative care needs in rural community care. This area is important to further investigate encompassing perspectives of patients, and their family members.

### Electronic supplementary material

Below is the link to the electronic supplementary material.

Supplementary Material 1

## Data Availability

Data are available upon reasonable request.

## Abbreviations

CRNs: Community registered nurses
HCPs: Healthcare professionals
PCT: Palliative consulting team
