# Supplementary material for: Healthcare professionals’ experiences of video consultations in palliative care in rural areas: an intervention study in community care
Source: BMC Health Serv Res. 2024 Jun 17;24:740. doi: 10.1186/s12913-024-11196-5 (PMC11181567; doi:10.1186/s12913-024-11196-5)
Supplement: Supplementary file 1 — Supplementary Material 1 [file 12913_2024_11196_MOESM1_ESM.docx]

**Interview guide**

**Background questions:**

Age? Gender? Education?

**Interview questions**

1. Can you share your experiences with video consultations in municipal home healthcare/telemedicine?
2. Can you describe a positive consultation experience?
3. Can you describe a less favorable consultation experience?
4. Do you find it challenging to discuss certain topics through video consultations?
5. What obstacles do you perceive in using video consultations?
6. What opportunities do you see in utilizing video consultations?
7. Are there any questions that are inappropriate/unethical to discuss via video consultations?

**Follow-up questions**

"How did you feel at that time?", "What did you do then?", and "What were you thinking at that moment?" Can you elaborate on this?
